# Linalool Reduces Virulence and Tolerance to Adverse Conditions of *Listeria monocytogenes*

**DOI:** 10.3390/antibiotics13060474

**Published:** 2024-05-22

**Authors:** Joel P. Dias, Fernanda C. Domingues, Susana Ferreira

**Affiliations:** CICS-UBI Health Sciences Research Centre, University of Beira Interior, Avenida Infante D. Henrique, 6200-506 Covilhã, Portugal; joel.pereira.dias@ubi.pt (J.P.D.); fcd@ubi.pt (F.C.D.)

**Keywords:** *Listeria monocytogenes*, linalool, virulence, quorum sensing, adverse conditions

## Abstract

*Listeria monocytogenes*, a foodborne pathogen causing listeriosis, poses substantial societal, economic, and public health challenges due to its resistance, persistence, and biofilm formation in the food industry. Exploring subinhibitory concentrations of compounds to target virulence inhibition and increase susceptibility to adverse conditions presents a promising strategy to mitigate its impact of *L. monocytogenes* and unveils new potential applications. Thus, this study aims to explore the effect of linalool on virulence factors of *L. monocytogenes* and potential use in the reduction in its tolerance to stressful conditions. This action was analysed considering the use of two sub-inhibitory concentrations of linalool, 0.312 and 0.625 mg/mL. We found that even with the lowest tested concentrations, a 65% inhibition of violacein production by *Chromobacterium violaceum*, 55% inhibition in biofilm formation by *L. monocytogenes* and 62% reduction on haemolysis caused by this bacterium were observed. In addition to its impact on virulence factors, linalool diminished the tolerance to osmotic stress (up to 4.3 log reduction after 24 h with 12% NaCl), as well as to high (up to 3.8 log reduction after 15 min at 55 °C) and low temperatures (up to 4.6 log reduction after 84 days with 12% NaCl at 4 °C). Thus, this study paves the way to further investigation into the potential utilization of linalool to mitigate the threat posed by *L. monocytogenes* in the field of food safety and public health.

## 1. Introduction

Foodborne diseases bring social, economic, and public health burden [1], with *Listeria monocytogenes* being one of these foodborne pathogens associated with human and animal diseases [2,3,4]. This bacterium causes listeriosis that has high impact in health of immunocompromised, elderly people and pregnant women. Severe infection can cause septicemia, meningitis, endocarditis and spontaneous abortion [5,6], leading to a high hospitalization and mortality rate [7,8]. This pathogen can survive adverse conditions, such as temperatures up to 45 °C, low pH, and high salt concentration and can proliferate at 4 °C [9,10,11]. These capabilities to survive in such conditions along with the various virulence factors, such as biofilm-forming capacity, motility and listeriolysin O [3,12] makes it a problem for the food industry and public health.

The use of natural compounds in the food industry has been highlighted, both to mitigate the harmful effects of foodborne diseases or in food preservation. In this context, they have been studied for their antimicrobial or even anti-virulence properties as alternatives to antibiotics, food preservatives or chemical disinfectants [13,14,15,16,17,18,19,20,21,22,23,24,25]. Various isolated compounds present in essential oils, herbs, and spices, such as carvacrol, cinnamaldehyde, eugenol and thymol have already shown bactericidal and inhibitory effects on toxin production, quorum sensing, motility and biofilm formation against diverse pathogenic bacteria, including *L. monocytogenes* [13,15,24,25,26,27]. Although some natural compounds have been extensively investigated, there is still a lack of studies concerning their potential as anti-virulence agents or their ability to enhance susceptibility to adverse conditions. One of such compounds is the terpene alcohol, linalool (C_10_H_18_O, 3,7-dimethyl-1,6-octadien-3-ol), which is a component of various essential oils and is generally recognized as safe (GRAS) by the Food and Drug Administration (FDA) [28]. This compound has already a high value global production market, mainly due to its numerous applications in various sectors, such as in cosmetology, food industry, production of household products or pharmaceutical industry [29]. Additionally, linalool is known by its bioactive properties, such as anti-inflammatory, antioxidant, anticancer, neuroprotective, antidepressant, hepatoprotective and antimicrobial activities [29,30]. Among the described antimicrobial activities, linalool has already demonstrated efficacy against both Gram-positive and Gram-negative bacteria, as well as fungi. Furthermore, the ability to inhibit virulence factors such as quorum sensing and biofilm formation by *L. monocytogenes*, *Acinetobacter baumannii* or *Candida albicans* has been demonstrated [29,31,32,33]. Despite the properties and potential of linalool in food industry, its physical characteristics can represent certain challenges, mainly associated with its solubility, volatility, and interactions with other components commonly found in the food industry [29].

Considering the mentioned above, we aimed with this work to evaluate the anti-virulence properties of linalool, considering *L. monocytogenes* motility, biofilm-forming ability or haemolytic capacity. Additionally, the effect of linalool on the tolerance of this bacterium to stress conditions (temperature and osmotic) usually found in the food sector were further evaluated.

## 2. Results

### 2.1. Effect of Linalool on Listeria monocytogenes Growth

**Figure 1:**
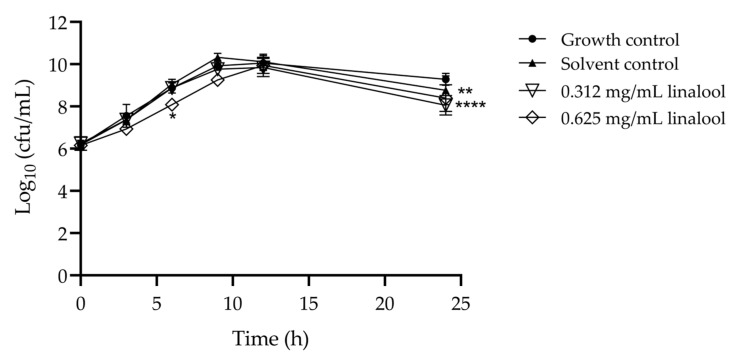
Growth curves for *L. monocytogenes* LMG 13305, incubated with 0.625 and 0.312 mg/mL of linalool, with growth and solvent controls. The results are presented as the mean ± standard deviation (SD). Asterisks represent significant differences to the growth control, * *p* < 0.05, ** *p* < 0.01, **** *p* < 0.0001 between the assay and the growth control.

The use of sub-inhibitory concentrations of compounds, such as linalool in this case, is necessary for assessing anti-virulence properties while avoiding significant effects on bacterial growth. Thus, the effect of subinhibitory concentrations of linalool in *L. monocytogenes* growth was evaluated (Figure 1). The solvent control (DMSO) had no significant reduction compared to the growth control. A slight reduction in growth can be observed at 0.625 mg/mL of linalool after 6 h of incubation and for both concentrations at 24 h. Given the minimal reduction observed in growth, we followed the assays with these concentrations.

### 2.2. Inhibition of Quorum Sensing in the Biosensor Bacterium Chromobacterium violaceum, and Motility and Biofilm Formation of Listeria monocytogenes, in the Presence of Linalool

**Figure 2:**
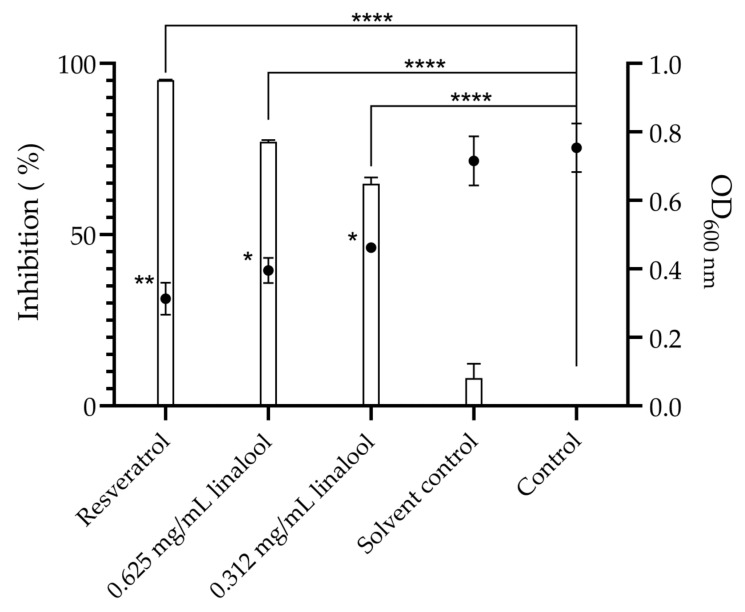
Quorum-sensing inhibition, represented by violacein percentage inhibition (columns) and microbial density (OD_600 nm_) (●) by 0.625 and 0.312 mg/mL of linalool, 19.5 µg/mL of resveratrol used as positive control and 0.06% of DMSO as a solvent control. The results are presented as the mean ± standard error of the means (SEM). Asterisks represent significant differences, in comparison to the control * (*p* < 0.05); ** (*p* < 0.01); **** (*p* < 0.0001).

Using a biosensor bacterium for assessing the effect of linalool as a quorum-sensing inhibitor, both defined concentrations of linalool demonstrated relevant inhibition of violacein production and subsequently quorum-sensing inhibition. Thus, the concentrations of 0.625 and 0.312 mg/mL of linalool showed a violacein production inhibition of 77.1% (*p* < 0.0001) and 64.9% (*p* < 0.0001), respectively, as well as for cell density (*p* < 0.01). The solvent (DMSO) showed no significant inhibition when compared with the control (Figure 2). 

**Figure 3:**
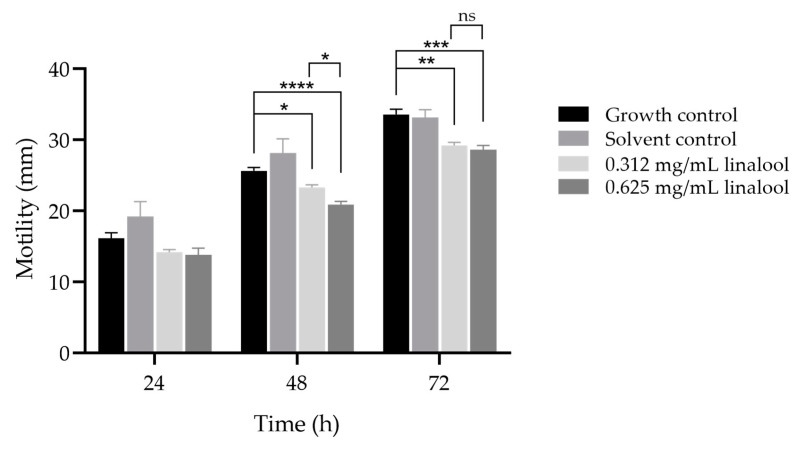
Inhibitory effect of 0.625 and 0.312 mg/mL of linalool in motility of *L. monocytogenes* LMG 13305, at 24, 48 and 72 h. The results are presented as the mean ± SD. ns (*p* > 0.05); * (*p* < 0.05); ** (*p* < 0.01); *** (*p* < 0.001); **** (*p* < 0.0001).

An inhibitory effect of linalool on *L. monocytogenes* motility was also observed for the subinhibitory concentrations tested, resulting in a significant reduction in the motility diameter for 48 and 72 h of incubation, when compared to the control (Figure 3), albeit modest. Furthermore, a significant difference was observed at 48 h between both concentrations of linalool used, although no differences were observed at other time points.

**Figure 4:**
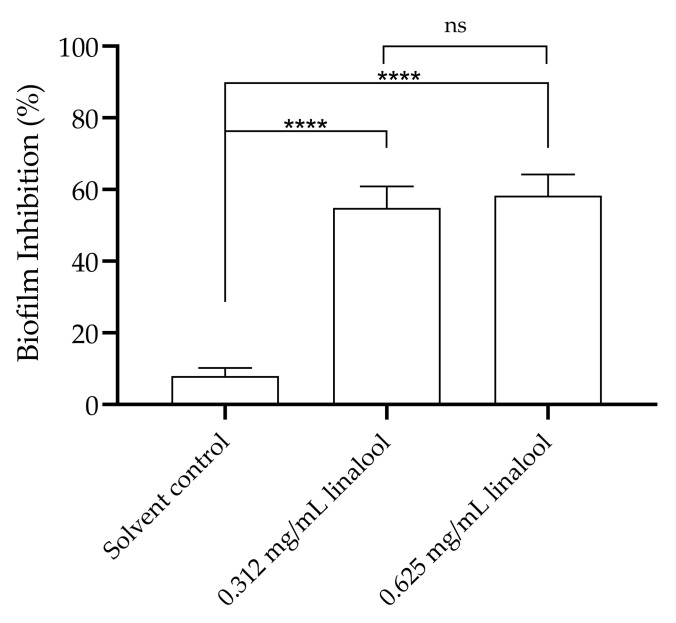
Inhibition of *L. monocytogenes* LMG 13305 biofilm formation by 0.625 and 0.312 mg/mL of linalool. The results are presented as the mean ± SEM. ns (*p* > 0.05); **** (*p* < 0.0001).

When analysing the influence of subinhibitory concentrations of linalool on biofilm formation, a significant reduction in the adhesion of *L. monocytogenes* and, therefore, in biofilm formation can be observed when compared to the solvent control (Figure 4). 

### 2.3. Study of the Haemolysis Capacity of Linalool in Humans’ Erythrocytes and Study of the Effect of Linalool in Haemolysis Induced by Listeria monocytogenes

**Figure 5:**
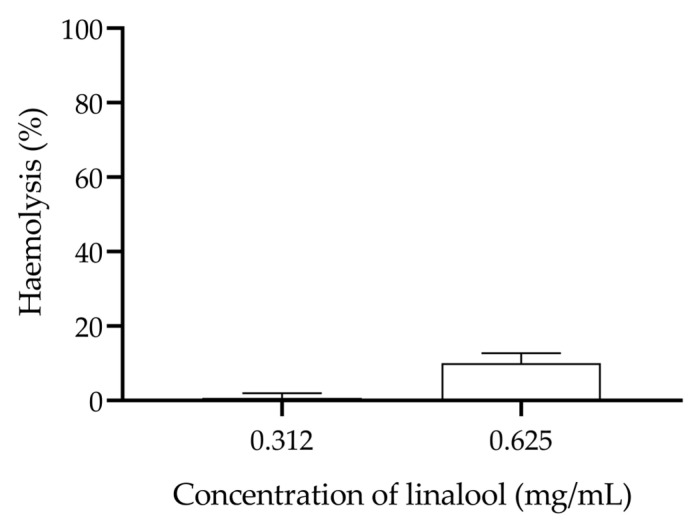
Evaluation of the haemolysis caused by different concentrations of linalool in humans’ erythrocytes.

Linalool showed low haemolytic activity against human erythrocytes at the subinhibitory concentrations studied in this work, with a maximum haemolysis of 10.1% at a concentration of 0.625 mg/mL (Figure 5).

**Figure 6:**
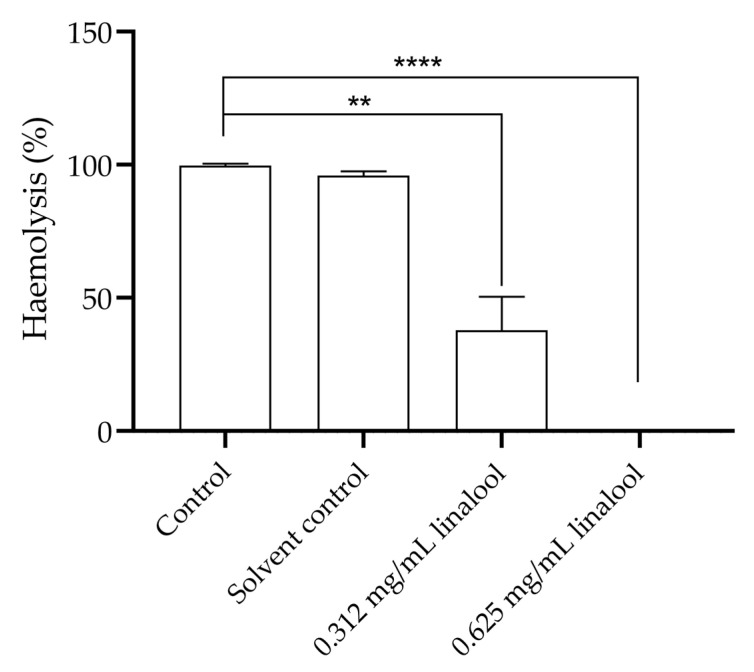
Haemolysis of humans’ erythrocytes by pre-exposure of *L. monocytogenes* LMG 13305 with 0.625 mg/mL and 0.312 mg/mL concentration of linalool. The results are presented as the mean ± SEM. Asterisks represent significant differences, in comparison to the control ** (*p* < 0.01); **** (*p* < 0.0001).

Pre-exposure of *L. monocytogenes* to 0.312 and 0.625 mg/mL of linalool was shown to lead to a significant reduction in the haemolysis of humans’ erythrocytes potentially caused by the Listeriolysin O (LLO) of *L. monocytogenes,* found in the culture supernatant (Figure 6). The concentration of 0.625 mg/mL showed a significant difference by completely inhibiting the haemolysis of human’s erythrocytes (*p* < 0.00001). The 0.312 mg/mL concentration reduced haemolysis to 37.8% when compared to the total haemolysis of erythrocytes that occurs in the control.

### 2.4. Evaluation of the Effect of Linalool in the Tolerance to Adverse Conditions

**Figure 7:**
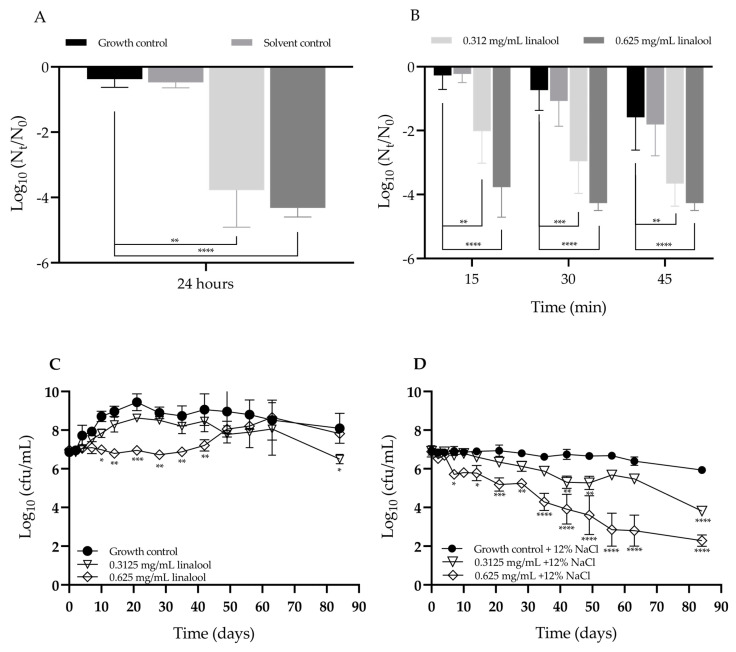
Evaluation of the effect of linalool on tolerance to: (**A**) osmotic stress (12% (*w*/*v*) NaCl); (**B**) high temperature (55 °C), (**C**) cold temperature (4 °C) and (**D**) cold temperature (4 °C) and 12% NaCl, by *L. monocytogenes* LMG 13305. The results are presented as the mean ± SD or SEM. Asterisks represent significant differences to the growth control, * *p* < 0.05, ** *p* < 0.01, *** *p* < 0.001, **** *p* < 0.0001 between the assay and the control.

When the tolerance to osmotic stress, in presence and absence of linalool, was tested, a significant decrease in survival of *L. monocytogenes* when in presence of 0.625 mg/mL and 0.312 mg/mL of linalool (Figure 7A) was observed. This behaviour contrasts from a 4.32 Log_10_ reduction in presence of 0.625 mg/mL of linalool to the one obtained for the control for which a Log_10_ reduction of 0.38 occurred.

When subjected to a high temperature of 55 °C, a significant decrease in survival of *L. monocytogenes* was observed when in presence of linalool comparing with the absence conditions, for all periods of incubation. The higher concentration of linalool had the most remarkable reduction in cell survival (Figure 7B). The lower concentration showed a significant reduction in cell viability but less noticeable in comparison to the previous concentration, ranging from a reduction in 2.02 log_10_ to 3.66 log_10_ from 15 to 45 min of incubation.

The cold temperature assays demonstrated a significant effect mainly of the 0.625 mg/mL concentration of linalool in the presence and absence of 12% NaCl at 4 °C (Figure 7C,D). There was also a difference between assays with and without NaCl. A significant decrease in the log count of *L. monocytogenes* from day 10 to 42 and at 84 days with the concentration of 0.625 mg/mL without NaCl and from day 14 onwards with 12% NaCl was observed.

## 3. Discussion

Natural compounds have shown in recent years to have noticeable antimicrobial and anti-virulence properties. Among these, linalool exhibits antimicrobial activity against a diversity of microorganisms and even anti-virulence properties, such as antibiofilm formation capacity [14,31,32,34]. 

Undoubtedly, *L. monocytogenes* continues to pose a significant public health threat due to its high mortality and hospitalization rates. This situation is exacerbated by the pathogen’s growing resistance to antimicrobial agents, in addition to its inherent resistance and adaptability to adverse environmental conditions encountered throughout food processing and storage, which allows it dissemination along the food chain [35,36]. In fact, the use of linalool has already been described due to its antilisterial properties [14,33,37,38]; however, these studies focused on evaluating its inhibitory activity rather than using subinhibitory concentrations of this compound to counteract the virulence traits or even modify the tolerance to stress conditions of this bacterium to other applications. Taking the above, deeper research about the anti-virulence properties and possible advantages of linalool against foodborne pathogens, such as *L. monocytogenes* is lacking.

Thus, this work focused on the potential impact of the natural compound, linalool, on the mitigation of virulence factors of *L. monocytogenes*, but also in the attenuation of tolerance to adverse conditions. For that, the subinhibitory concentrations of linalool of 0.625 mg/mL and 0.312 mg/mL were used, ensuring that the selected concentrations would not hinder growth, as confirmed by the growth curves assay (Figure 1). 

Considering quorum sensing as a mechanism used by bacteria to coordinate communal behaviour and that can regulate bacterial population’s physiological features or even virulence factors, such as motility, biofilm formation, extracellular enzyme secretion amongst others [39,40,41], the effect of linalool on this communication process was assessed. *C. violaceum* is commonly used as a biosensor organism for studying quorum-sensing inhibition due to its sensitivity to quorum sensing signals and the ease of monitoring violacein production [41]. Based on the results, linalool appears to have an anti-quorum sensing activity, due to the strong inhibition of violacein production observed in linalool’s presence (Figure 2). In fact, this anti-quorum-sensing inhibition was previously suggested in other studies using *C. violaceum* as a biosensor, but also in *P. aeruginosa* [31,40]. Despite a certain amount of inhibition being associated with a reduction in cell density, the inhibition of violacein is not only associated with this trend. A possible explanation for this is the ability of linalool to downregulate violacein biosynthetic genes and of attenuating quorum sensing by controlling the cell density through *cviI/R* downregulation [41]. In turn, a two-hours exposure of *L. monocytogenes* to linalool led to an increase in the expression of genes participating in quorum sensing, thus activating this mechanism, while inhibiting motility and chemotaxis [37].

In line with the role of quorum sensing as a signalling mechanism in bacteria that is associated with the modulation of virulence mechanisms and pathogenicity, later in this study, motility and biofilm formation were investigated. These features were also considered as a key factor in the surface colonization, increased resistance to antimicrobial agents, survival and persistence of *L. monocytogenes* [42,43,44]. Consistent with findings in other bacteria, a decrease in *L. monocytogenes* motility was observed in the presence of linalool, which may be associated with a down-regulation of FliN by linalool treatment, which may hinder flagellar synthesis and motility [37]. A study has shown that *Cinnamomum camphora* essential oil, in which linalool is one of the main components, inhibited motility in *C. violaceum* [42], while isolated linalool inhibited swarming and swimming motility of *Vibrio harveyi* [45]. Likely associated with the observed inhibition of quorum sensing and motility, a significant inhibitory effect on *L. monocytogenes* biofilm formation was also found with both subinhibitory concentrations of linalool tested. This phenomenon aligns with findings in other microorganisms, such as *Acinetobacter baumannii*, *Shigella flexneri*, *Candida albicans*, and *Pseudomonas aeruginosa* [31,46,47,48]. Moreover, previously, other study demonstrated that linalool at minimum inhibitory concentration could eradicate a 72-h-old *L. monocytogenes*’ biofilm. The authors propose that this eradication may be attributed to the formation hollows and holes in the biofilm by linalool, as well as a reduction in the biofilm thickness upon treatment with linalool [33]. In our work, the observed effect may be due to inhibition of energy acquisition pathways or action on specific targets or key enzymes [29,37,38], rather than structural biofilm modifications. This could potentially explain the lack of difference in effect between the two tested concentrations. The consistency between the results of this study and previous findings suggests the potential of linalool in inhibiting biofilm formation and reducing the persistence of *L. monocytogenes* in the food industry. 

Associated with the pathogenicity of *L. monocytogenes*, is Listeriolysin O (LLO), a pH-dependent haemolysin. LLO facilitates internalization in phagosomes and erythrocytes, allowing the bacteria to escape through membrane rupture and release into the cytoplasm [49,50]. For this reason, LLO and haemolysis are key virulence factors of *L. monocytogenes*. Given that LLO activity may be detected through an in vitro haemolysis assay [51], we initiated with an assessment of linalool-induced haemolysis in erythrocytes, followed by an evaluation of the inhibition of haemolysis by pre-incubation of *L. monocytogenes* with linalool. Our research findings indicated that subinhibitory concentrations of linalool showed low haemolytic activity. However, a notable decrease in the haemolytic activity of *L. monocytogenes* was observed following pre-exposure to linalool, suggesting that it likely directly targets the LLO, thereby reducing the haemolytic activity of the bacterium, which will attenuate the impact of this virulence factor. Indeed, linalool have shown to act through different mechanisms and targeting different structures, such as cell membranes, cell walls, nucleoids, and ribosomes [14,33], being able to disrupt cellular function and lead to cell death by acting on the cell membrane, but also inhibiting pathways related with energy obtention, leading in turn to metabolic disfunctions and inhibition of key enzymes [29,37,38].

*L. monocytogenes* exhibits remarkable resilience, thriving in adverse conditions and adapt to environmental stress commonly encountered in food production or storage, such as temperature or osmotic stress. Therefore, we further assessed how linalool could affect the susceptibility of this bacterium to such adverse conditions. Heat treatment, a method commonly used in food processing to preserve and prevent contamination [47], has sometimes be shown ineffective due to the resistance of *L. monocytogenes* to temperatures above 45 °C [52]. Hence, we evaluated the impact of the subinhibitory concentrations of linalool on the bacterium’s tolerance to 55 °C for 45 min, noting a significant reduction in survival at all time points tested, indicating a potentiation of the effect of temperature on the *L. monocytogenes.* Considering that *L. monocytogenes* is a successful bacterium in adapting to stress conditions, having the capacity to thrive in environments containing on average 12 to 20% NaCl [53], its osmoadaptation in the presence of linalool was studied. It was found that in the presence of linalool, the strain became more susceptible to osmotic stress. 

The ability of *L. monocytogenes* to survive and succeed at low temperatures is well described [54,55]. This growth ability at low temperatures for long period times was observed in this work, with this bacterium surviving to low temperatures and 12% of NaCl for at least 84 days, simulating long-term storage conditions relevant to certain environmental or industrial scenarios. Nonetheless, when in the presence of subinhibitory concentrations of linalool, a reduction in growth or even increase in death was observed at a cold temperature or cold temperature and osmotic stress, respectively. 

In fact, linalool showed the capacity to increase susceptibility to all the adverse conditions tested, which may be correlated to the capability of linalool to target cell membranes and walls [14,29] and thus acting synergistically with temperature and osmotic stresses, or influence the cell metabolism, targeting nucleoids and ribosomes [14] and thus making it harder for *L. monocytogenes* to adapt to the adverse conditions. The capacity of linalool to enhance the susceptibility of *L. monocytogenes* to various adverse conditions underscores the potential efficacy of linalool in preventing food contamination, in addition to its ability to reduce the virulence of this foodborne pathogen.

## 4. Materials and Methods

### 4.1. Linalool, Bacterial Strain and Growth Conditions

Linalool, (±)-3,7-dimethyl-3-hydroxy-1,6-octadiene), ((CH_3_)_2_C=CHCH_2_CH_2_C(CH_3_)(OH)CH=CH_2_) was purchased at Sigma-Aldrich (L2602, St. Louis, MO, USA). All the assays were performed using two linalool subinhibitory concentrations (0.625 mg/mL and 0.312 mg/mL). Its antimicrobial activity was evaluated against *Listeria monocytogenes* LMG 13305 (serotype 4b) from BCCM/LMG collection (Ghent, Belgium). The strain was cultured in Tryptic soy agar (TSA, VWR International, Leuven, Belgium) at 37 °C for 24 h prior to testing. 

### 4.2. Growth Curves Determination in Presence of Linalool

For the determination of the time-kill curves, first, a culture was prepared by suspending a colony of *L. monocytogenes* in 10 mL of Tryptic soy broth (TSB) and incubating at 37 °C for 16 h at 250 rpm. After the overnight incubation, the inoculum was diluted to a final cell concentration of about 10^6^ cfu/mL and exposed to linalool concentrations of 0.625 and 0.312 mg/mL (DMSO at 0.5% (*v*/*v*)). Solvent and growth controls were performed. The cultures were incubated at 37 °C for 24 h and cell counts were made at 0, 3, 6, 9, 12 and 24 h, by drop-plate method. This experiment was performed three independent times.

### 4.3. Quorum-Sensing Inhibition by Linalool

For this assay, the biosensor bacterium *Chromobacterium violaceum* ATCC 12472 was used to evaluate the effect of linalool in quorum sensing [15,56]. An overnight culture of *C. violaceum* was prepared at 30 °C, 250 rpm for 16 h with Luria Bertani medium (LB). After the incubation, in a 48-wells plate, the bacterial suspension was exposed to 0.625 and 0.312 mg/mL of linalool at a final cell concentration of ~1 × 10^6^ cfu/mL, in a volume of 1 mL by well. A growth control, solvent control (DMSO 0.06% (*v*/*v*)) and control of quorum-sensing inhibition (resveratrol at 19.5 µg/mL) were performed [15]. The plate was incubated for 48 h at 30 °C. For the evaluation of violacein inhibition, after the incubation, 750 µL of each well was centrifuged at 6800× *g* for 3 min. The pellet was then suspended in 750 µL of DMSO for extracting violacein. This suspension was then centrifuged at 8000× *g* for 5 min to obtain a supernatant with violacein and a pellet of bacterial cells. From this supernatant, 200 µL were collected in triplicate to a 96-well plate for the reading of absorbance at 585 nm. To assess if inhibition of violacein could occur due to the bacterial inhibition, the pellet was then resuspended in 750 µL of distilled water and the optical density at 600 nm was read. Three independent assays were performed in triplicate. The percentage of violacein inhibition was calculated through the formula:

(1) $$\;\mathrm{Violacein}\;\mathrm{inhibition}\;(\%)\;=100\;-\left(\left(\frac{\;\mathrm{sample}\;OD_{585}}{\;\mathrm{Growth}\;\mathrm{control}\;OD_{585}}\right)\right)\times 100$$

### 4.4. Evaluation of Listeria monocytogenes Motility When Exposed to Linalool 

The effect of linalool on the motility of *L. monocytogenes* LMG 13305 was assessed as described by [25]. From the overnight culture, the inoculum was adjusted to a OD_600 nm_ = 1 (~10^9^ cfu/mL). Then, 5 µL of this suspension was pipetted to TSB with 0.3% (*w*/*v*) of agar and 0.625 or 0.312 mg/mL of linalool. Motility control and solvent control with a 0.128% (*v*/*v*) concentration of DMSO were also included. The plates were incubated for 72 h at 30 °C, and the motility diameter measured in millimetres (mm) at 24, 48 and 72 h. This experiment was performed four independent times.

### 4.5. Inhibition of Listeria monocytogenes Biofilm Formation in the Presence of Linalool

The analysis of the effect of linalool in biofilm formation by *L. monocytogenes* was performed based on [15,57] with modifications, such as the use of 48-well plates. After an overnight culture, the inoculum was adjusted to a OD_600 nm_ = 0.1 (~10^8^ cfu/mL) and was subsequently added to a 48-well plate together with the compound’s solution, thus reaching a final concentration 0.625 and 0.312 mg/mL of linalool, to a cell density of ~10^7^ cfu/mL on a 500 µL final volume. Wells of solvent (DMSO at 0.06% (*v*/*v*)) and growth control were also made. The plates were incubated for 24 h at 37 °C. After the incubation, the wells were carefully washed twice with 500 µL of distilled water and then fixed with 500 µL of methanol for 20 min. The methanol was removed and, after the plates had dried, 0.1% (*w*/*v*) crystal violet was added and left to rest for 10 min. After this time, the crystal violet was removed, and the wells were washed three times with distilled water. Then, 500 μL of 33% (*v*/*v*) acetic acid was added to dissolve the dye and finally the absorbance was read at 570 nm. Four replicates were made six independent times. The percentage of biofilm formation inhibition was calculated considering the control.

### 4.6. Haemolytic Activity of Linalool in Humans’ Erytrocytes 

The haemolysis caused by linalool on human erythrocytes was analysed, as previously described [58]. For this, in a 96-well plate with U bottom, serial dilutions of linalool in PBS were prepared (a range of concentrations from 2.5 to 0.312 mg/mL, with a maximum of 0.5% (*v*/*v*) of DMSO) and 100 µL of human erythrocytes obtained from blood collected from a healthy volunteer from the research team at 2% (*v*/*v*) were added, achieving a final volume of 200 µL in each well. The 96-well plate was then incubated for 24 h at 37 °C. After this period, the plates were centrifuged at 1000× *g* for 5 min and the supernatant was collected into another 96-well plate to be read at 543 nm. A negative control (PBS) and a positive control (1% (*v*/*v*) Triton X 100) were also performed. This assay was completed three independent times.

### 4.7. Study of the Inhibitory Capabilities of Linalool on Human Erythrocytes Haemolysis Caused by Listeria monocytogenes

The inhibition of haemolysis by *Listeria monocytogenes* was assessed as previously described [59], with minor modifications. *Listeria monocytogenes* LMG 13305 was incubated overnight at 37 °C with shaking, and then subcultured in TSB containing 0.625 and 0.312 mg/mL of linalool and a final cell concentration of 10^6^ CFU/mL, for 10 h at 37 °C. After this period, the cultures were centrifuged at 9000× *g* at 4 °C for 10 min, from which 100 µL of supernatant were obtained and added to 100 µL of erythrocytes 2% (*v*/*v*) and incubated for 30 min at 37 °C. After the incubation, the plate was centrifuged at 1000× *g* for 5 min. Then, 100 µL of the supernatant was removed from each well to a new 96-well plate to be read at 543 nm. A solvent control, positive control (supernatant from non-exposed bacteria), negative control (without bacterial growth) and a positive control of total haemolysis (1% (*v*/*v*) Triton X 100) were also included. This assay was performed three independent times.

### 4.8. Evaluation of Linalool on the Tolerance of Listeria monocytogenes to Adverse Conditions 

The effect of linalool on the tolerance of *L. monocytogenes* to adverse conditions, such as osmotic stress, high and low temperatures, was assessed [25,60]. A bacterial suspension was prepared from the overnight culture and adjusted to an OD_600 nm_ = 0.1 (~10^8^ cfu/mL). Then, 30 µL was added to tubes with 2970 µL of TSB medium, TSB with DMSO (0.06% (*v*/*v*), or with 0.625 or 0.312 mg/mL of linalool.

(i) To assess the effect on osmotic stress, the tubes were supplemented with NaCl at 12% (*w*/*v*). The tubes were incubated for 24 h and measurements were made at 0 and 24 h.
(ii) The effect of a high temperature was evaluated by incubating tubes at 55 °C, and samples collected at 0, 15, 30 and 45 min.
(iii) The effect of low temperature was assessed also in absence or presence of osmotic stress; thus, the experiment was conducted using TSB or TSB with 12% NaCl (*w*/*v*), respectively; in this case, in a final volume of 1 mL. The tubes were incubated at a low storage temperature of 4 °C for 84 days. The viable count measurements were performed by drop-plate methods, and the assays repeated at least two independent times.

### 4.9. Statistical Analysis

The Student’s *t*-test was used for statistical analysis by using GraphPad Prism v8.0.2 software. A two-way ANOVA, with a Tukey multiple comparison test, was performed to test the levels of bacteria cell counts for growth curves, and when testing linalool at cold and cold with osmotic stress conditions. Significant differences (*p* < 0.05) are indicated by asterisks in all figures, with * *p* < 0.05, ** *p* < 0.01, *** *p* < 0.001, **** *p* < 0.0001.

## 5. Conclusions

Subinhibitory concentrations of linalool were found to effectively counteract virulence of *L. monocytogenes* by targeting key factors, such as quorum sensing, motility, biofilm formation and haemolysis. In addition, it may increase the susceptibility of *L. monocytogenes* to adverse conditions, such as those typically used in the food industry. In sum, these results highlight the potential of linalool even at lower concentrations to overcome some of the problems posed by *L. monocytogenes* in the field of food safety. Nonetheless, more studies are needed to validate the application of this compound in the food industry, namely through the inclusion of strains isolated from food or evaluation of the potential interaction of linalool with food components.

## Data Availability

Data is contained within the manuscript.

## References

[1] Food Safety. https://www.who.int/news-room/fact-sheets/detail/food-safety.

[2] Shamloo E., Hosseini H., Moghadam A.Z., Larsen H.M., Haslberger A., Alebouyeh M. (2019). Importance of *Listeria monocytogenes* in food safety: A review of its prevalence, detection, and antibiotic resistance. Iran. J. Vet. Res..

[3] Kannan S., Balakrishnan J., Govindasamy A. (2020). *Listeria monocytogens*—Amended understanding of its pathogenesis with a complete picture of its membrane vesicles, quorum sensing, biofilm and invasion. Microb. Pathog..

[4] Freitag N.E., Port G.C., Miner M.D. (2009). *Listeria monocytogenes*—From saprophyte to intracellular pathogen. Nat. Rev. Microbiol..

[5] McDougal C.E., Sauer J.D. (2018). *Listeria monocytogenes:* The Impact of Cell Death on Infection and Immunity. Pathogens.

[6] Tricoli M.R., Massaro C., Arrigo I., Diquattro O., Di Bernardo F., Galia E., Palermo M., Fasciana T., Giammanco A. (2024). Characterization of *Listeria monocytogenes* Strains Isolated in Palermo (Sicily and Italy) during the Years 2018–2020 from Severe Cases of Listeriosis. Antibiotics.

[7] European Food Safety Authority (EFSA), European Centre for Disease Prevention and Control (ECDC) (2021). The European Union One Health 2019 Zoonoses Report. EFSA J..

[8] Lomonaco S., Nucera D., Filipello V. (2015). The evolution and epidemiology of *Listeria monocytogenes* in Europe and the United States. Infect. Genet. Evol..

[9] Hernandez-Milian A., Payeras-Cifre A. (2014). What is new in Listeriosis?. Biomed. Res. Int..

[10] Todd E.C.D., Notermans S. (2011). Surveillance of listeriosis and its causative pathogen, *Listeria monocytogenes*. Food Control.

[11] Duze S.T., Marimani M., Patel M. (2021). Tolerance of *Listeria monocytogenes* to biocides used in food processing environments. Food Microbiol..

[12] Matle I., Mbatha K.R., Madoroba E. (2020). A review of *Listeria monocytogenes* from meat and meat products: Epidemiology, virulence factors, antimicrobial resistance and diagnosis. Onderstepoort J. Vet. Res..

[13] Silva L.N., Zimmer K.R., Macedo A.J., Trentin D.S. (2016). Plant Natural Products Targeting Bacterial Virulence Factors. Chem. Rev..

[14] Gao Z., Zhong W., Liu T., Zhao T., Guo J. (2021). Global proteomic analysis of *Listeria monocytogenes*’ response to linalool. Foods.

[15] Carvalho F., Coimbra A.T., Silva L., Duarte A.P., Ferreira S. (2023). *Melissa officinalis* essential oil as an antimicrobial agent against *Listeria monocytogenes* in watermelon juice. Food Microbiol..

[16] Fasciana T., Gargano M.L., Serra N., Galia E., Arrigo I., Tricoli M.R., Diquattro O., Graceffa G., Vieni S., Venturella G. (2021). Potential activity of albino *Grifola frondosa* mushroom extract against biofilm of meticillin-resistant *Staphylococcus aureus*. J. Fungi.

[17] Gargano M.L., Zervakis G.I., Isikhuemhen O.S., Venturella G., Calvo R., Giammanco A., Fasciana T., Ferraro V. (2020). Ecology, Phylogeny, and Potential Nutritional and Medicinal Value of a Rare White “Maitake” Collected in a Mediterranean Forest. Diversity.

[18] Abdelhamid A.G., El-Dougdoug N.K. (2020). Controlling foodborne pathogens with natural antimicrobials by biological control and antivirulence strategies. Heliyon.

[19] Lu G., Xu L., Zhang P., Dou X., Yu J., Feng H. (2019). Betulin efficiently suppresses the process of an experimental *Listeria monocytogenes* infection as an antagonist against listeriolysin O. Fitoterapia.

[20] Gowrishankar S., Sivaranjani M., Kamaladevi A., Ravi A.V., Balamurugan K., Karutha Pandian S. (2016). Cyclic dipeptide cyclo(l-leucyl-l-prolyl) from marine *Bacillus amyloliquefaciens* mitigates biofilm formation and virulence in *Listeria monocytogenes*. Pathog. Dis..

[21] Liu M., Lv Q., Xu J., Liu B., Zhou Y., Zhang S., Shen X., Wang L. (2023). Isoflavone glucoside genistin, an inhibitor targeting Sortase A and Listeriolysin O, attenuates the virulence of *Listeria monocytogenes* in vivo and in vitro. Biochem. Pharmacol..

[22] Nwabor O.F., Vongkamjan K., Voravuthikunchai S.P. (2019). Antioxidant Properties and Antibacterial Effects of *Eucalyptus camaldulensis* Ethanolic Leaf Extract on Biofilm Formation, Motility, Hemolysin Production, and Cell Membrane of the Foodborne Pathogen *Listeria monocytogenes*. Foodborne Pathog. Dis..

[23] Song M., Teng Z., Li M., Niu X., Wang J., Deng X. (2017). Epigallocatechin gallate inhibits *Streptococcus pneumoniae* virulence by simultaneously targeting pneumolysin and sortase A. J. Cell. Mol. Med..

[24] Wang J., Qiu J., Tan W., Zhang Y., Wang H., Zhou X., Liu S., Feng H., Li W., Niu X. (2015). Fisetin Inhibits *Listeria monocytogenes* Virulence by Interfering with the Oligomerization of Listeriolysin O. J. Infect. Dis..

[25] Coimbra A., Carvalho F., Duarte A.P., Ferreira S. (2022). Antimicrobial activity of *Thymus zygis* essential oil against *Listeria monocytogenes* and its application as food preservative. Innov. Food Sci. Emerg. Technol..

[26] Zhang D., Gan R.Y., Zhang J.R., Farha A.K., Li H.B., Zhu F., Wang X.H., Corke H. (2020). Antivirulence properties and related mechanisms of spice essential oils: A comprehensive review. Compr. Rev. Food Sci. Food Saf..

[27] Awad A.H.R., Parmar A., Ali M.R., El-Mogy M.M., Abdelgawad K.F. (2021). Extending the shelf-life of fresh-cut green bean pods by ethanol, ascorbic acid, and essential oils. Foods.

[28] CFR—Code of Federal Regulations Title 21. Food and Drugs, Chapter I-Food and Drug Administration, Department of Health and Human Services, Subchapter B—Food for Human Consumption, Part 182-Substances Generally Recognized as Safe, Sec. 182.60 Synthetic. https://www.accessdata.fda.gov/scripts/cdrh/cfdocs/cfCFR/CFRSearch.cfm?fr=182.60&SearchTerm=linalool.

[29] Mączka W., Duda-Madej A., Grabarczyk M., Wińska K. (2022). Natural Compounds in the Battle against Microorganisms—Linalool. Molecules.

[30] An Q., Ren J.N., Li X., Fan G., Qu S.S., Song Y., Li Y., Pan S.Y. (2021). Recent updates on bioactive properties of linalool. Food Funct..

[31] Alves S., Duarte A., Sousa S., Domingues F.C. (2016). Study of the major essential oil compounds of *Coriandrum sativum* against *Acinetobacter baumannii* and the effect of linalool on adhesion, biofilms and quorum sensing. Biofouling.

[32] Manoharan R.K., Lee J.H., Kim Y.G., Kim S.-I., Lee J. (2017). Inhibitory effects of the essential oils α-longipinene and linalool on biofilm formation and hyphal growth of *Candida albicans*. Biofouling.

[33] Gao Z., Van Nostrand J.D., Zhou J., Zhong W., Chen K., Guo J. (2019). Anti-listeria Activities of Linalool and Its Mechanism Revealed by Comparative Transcriptome Analysis. Front. Microbiol..

[34] Fisher K., Phillips C.A. (2006). The effect of lemon, orange and bergamot essential oils and their components on the survival of *Campylobacter jejuni*, *Escherichia coli* O157, *Listeria monocytogenes*, *Bacillus cereus* and *Staphylococcus aureus* in vitro and in food systems. J. Appl. Microbiol..

[35] Lopes-Luz L., Mendonça M., Fogaça B., Kipnis A., Bhunia A.K., Bührer-Sékula S., Mendonc¸a M., Bernardes Fogaça M., Kipnis A.E., B€ Uhrer-S Ekula S. (2021). *Listeria monocytogenes*: Review of pathogenesis and virulence determinants-targeted immunological assays. Crit. Rev. Microbiol..

[36] Matereke L.T., Okoh A.I. (2020). *Listeria monocytogenes* Virulence, Antimicrobial Resistance and Environmental Persistence: A Review. Pathogens.

[37] He R., Zhong Q., Chen W., Zhang M., Pei J., Chen H., Chen W. (2023). Transcriptomic and proteomic investigation of metabolic disruption in *Listeria monocytogenes* triggered by linalool and its application in chicken breast preservation. LWT.

[38] He R., Chen W., Chen H., Zhong Q., Zhang H., Zhang M., Chen W. (2022). Antibacterial mechanism of linalool against *L. monocytogenes*, a metabolomic study. Food Control.

[39] Abisado R.G., Benomar S., Klaus J.R., Dandekar A.A., Chandler J.R. (2018). Bacterial quorum sensing and microbial community interactions. MBio.

[40] Lahiri D., Nag M., Dutta B., Dey S., Mukherjee D., Joshi S.J., Ray R.R. (2021). Antibiofilm and anti-quorum sensing activities of eugenol and linalool from *Ocimum tenuiflorum* against *Pseudomonas aeruginosa* biofilm. J. Appl. Microbiol..

[41] Kanekar S., Devasya R.P. (2022). Growth-phase specific regulation of *cviI/R* based quorum sensing associated virulence factors in Chromobacterium violaceum by linalool, a monoterpenoid. World J Microbiol Biotechnol..

[42] Wang W., Li D., Huang X., Yang H., Qiu Z., Zou L., Liang Q., Shi Y., Wu Y., Wu S. (2019). Study on Antibacterial and Quorum-Sensing Inhibition Activities of *Cinnamomum camphora* Leaf Essential Oil. Molecules.

[43] Zhang Y., Kong J., Xie Y., Guo Y., Cheng Y., Qian H., Yao W. (2018). Essential oil components inhibit biofilm formation in *Erwinia carotovora* and *Pseudomonas fluorescens* via anti-quorum sensing activity. LWT.

[44] Carpentier B., Cerf O. (2011). Review—Persistence of *Listeria monocytogenes* in food industry equipment and premises. Int. J. Food Microbiol..

[45] Sowndarya J., Farisa Banu S., Madhura G., Yuvalakshmi P., Rubini D., Bandeira Junior G., Baldisserotto B., Vadivel V., Nithyanand P. (2019). Agro food by-products and essential oil constituents curtail virulence and biofilm of *Vibrio harveyi*. Microb. Pathog..

[46] Ngome M.T., Alves J.G., de Oliveira A.C., da Silva Machado P., Mondragón-Bernal O.L., Piccoli R.H. (2018). Linalool, citral, eugenol and thymol: Control of planktonic and sessile cells of *Shigella flexneri*. AMB Express.

[47] Hsu C.C., Lai W.L., Chuang K.C., Lee M.H., Tsai Y.C. (2013). The inhibitory activity of linalool against the filamentous growth and biofilm formation in *Candida albicans*. Med. Mycol..

[48] Kim Y.G., Lee J.H., Kim S.I., Baek K.H., Lee J. (2015). Cinnamon bark oil and its components inhibit biofilm formation and toxin production. Int. J. Food Microbiol..

[49] Schnupf P., Portnoy D.A. (2007). Listeriolysin O: A phagosome-specific lysin. Microbes Infect..

[50] Vadia S., Arnett E., Haghighat A.C., Wilson-Kubalek E.M., Tweten R.K., Seveau S. (2011). The Pore-Forming Toxin Listeriolysin O Mediates a Novel Entry Pathway of *L. monocytogenes* into Human Hepatocytes. PLoS Pathog..

[51] Liu Z., Meng R., Zhao X., Shi C., Zhang X., Zhang Y., Guo N. (2016). Inhibition effect of tea tree oil on *Listeria monocytogenes* growth and exotoxin proteins listeriolysin O and p60 secretion. Lett. Appl. Microbiol..

[52] Arioli S., Montanari C., Magnani M., Tabanelli G., Patrignani F., Lanciotti R., Mora D., Gardini F. (2019). Modelling of *Listeria monocytogenes* Scott A after a mild heat treatment in the presence of thymol and carvacrol: Effects on culturability and viability. J. Food Eng..

[53] Bucur F.I., Grigore-Gurgu L., Crauwels P., Riedel C.U., Nicolau A.I. (2018). Resistance of *Listeria monocytogenes* to Stress Conditions Encountered in Food and food processing environments. Front. Microbiol..

[54] Tasara T., Stephan R. (2006). Cold Stress Tolerance of *Listeria monocytogenes*: A Review of Molecular Adaptive Mechanisms and Food Safety Implications. J. Food Prot..

[55] Cabrita P., Trigo M.J., Ferreira R.B., Brito L. (2015). Differences in the Expression of Cold Stress–Related Genes and in the Swarming Motility Among Persistent and Sporadic Strains of *Listeria monocytogenes*. Foodborne Pathog. Dis..

[56] Asensio C.M., Quiroga P.R., Al-Gburi A., Huang Q., Grosso N.R. (2020). Rheological Behavior, Antimicrobial and Quorum Sensig Inhibition Study of an Argentinean Oregano Essential Oil Nanoemulsion. Front. Nutr..

[57] Stepanović S., Ćirković I., Ranin L., Švabić-Vlahović M. (2004). Biofilm formation by *Salmonella* spp. and Listeria monocytogenes on plastic surface. Lett. Appl. Microbiol..

[58] Duarte A.F., Ferreira S., Oliveira R., Domingues F.C. (2013). Effect of Coriander Oil (*Coriandrum sativum*) on Planktonic and Biofilm Cells of *Acinetobacter baumannii*. Nat. Prod. Commun..

[59] Du W., Zhou M., Liu Z., Chen Y., Li R. (2018). Inhibition effects of low concentrations of epigallocatechin gallate on the biofilm formation and hemolytic activity of *Listeria monocytogenes*. Food Control.

[60] Oliveira A.R., Domingues F.C., Ferreira S. (2017). The influence of resveratrol adaptation on resistance to antibiotics, benzalkonium chloride, heat and acid stresses of *Staphylococcus aureus* and *Listeria monocytogenes*. Food Control.

